# Subtilomycin: A New Lantibiotic from *Bacillus subtilis* Strain MMA7 Isolated from the Marine Sponge *Haliclona simulans*

**DOI:** 10.3390/md11061878

**Published:** 2013-06-03

**Authors:** Robert W. Phelan, Matthieu Barret, Paul D. Cotter, Paula M. O’Connor, Rui Chen, John P. Morrissey, Alan D. W. Dobson, Fergal O’Gara, Teresa M. Barbosa

**Affiliations:** 1 Department of Microbiology, University College Cork, Cork, Ireland; E-Mails: 105406340@umail.ucc.ie (R.W.P.); matthieu.barret@angers.inra.fr (M.B.); j.morrissey@ucc.ie (J.P.M.); a.dobson@ucc.ie (A.D.W.D.); 2 Biomerit Research Centre, Department of Microbiology, University College Cork, Cork, Ireland; 3 Teagasc Food Research Centre, Teagasc, Moorepark, Fermoy, Co. Cork, Ireland; E-Mails: P.Cotter@ucc.ie (P.D.C.); Paula.OConnor@teagasc.ie (P.M.O.); 4 Alimentary Pharmabiotic Centre, University College Cork, Cork, Ireland; 5 Department of Molecular and Human Genetics, Baylor College of Medicine, Houston, TX 77030, USA; E-Mail: ruichen@bcm.edu; 6 Marine Biotechnology Centre, Environmental Research Institute, University College Cork, Cork, Ireland; 7 School of Pharmacy, University College Cork, Cork, Ireland

**Keywords:** antimicrobial, subtilomycin, lantibiotic, marine sponge, *Bacillus subtilis*

## Abstract

Bacteriocins are attracting increased attention as an alternative to classic antibiotics in the fight against infectious disease and multidrug resistant pathogens. *Bacillus subtilis* strain MMA7 isolated from the marine sponge *Haliclona simulans* displays a broad spectrum antimicrobial activity, which includes Gram-positive and Gram-negative pathogens, as well as several pathogenic *Candida* species. This activity is in part associated with a newly identified lantibiotic, herein named as subtilomycin. The proposed biosynthetic cluster is composed of six genes, including protein-coding genes for LanB-like dehydratase and LanC-like cyclase modification enzymes, characteristic of the class I lantibiotics. The subtilomycin biosynthetic cluster in *B. subtilis* strain MMA7 is found in place of the sporulation killing factor (*skf*) operon, reported in many *B. subtilis* isolates and involved in a bacterial cannibalistic behaviour intended to delay sporulation. The presence of the subtilomycin biosynthetic cluster appears to be widespread amongst *B. subtilis* strains isolated from different shallow and deep water marine sponges. Subtilomycin possesses several desirable industrial and pharmaceutical physicochemical properties, including activity over a wide pH range, thermal resistance and water solubility. Additionally, the production of the lantibiotic subtilomycin could be a desirable property should *B. subtilis* strain MMA7 be employed as a probiotic in aquaculture applications.

## 1. Introduction

The quest for novel antimicrobial compounds from the marine environment has been driven by a widespread resistance to antibiotics and emerging multi drug resistant pathogens. The large majority of the antimicrobials currently used in clinical therapy are, or are derived from, natural products of microbial origin [1]. While antimicrobials have been traditionally isolated from soil-dwelling microorganisms, the number of bioactive metabolites isolated from marine organisms, and in particular from marine sponges, has increased dramatically in recent years [2,3,4].

Marine sponges (*Porifera*) are simple multicellular, sessile filter feeding invertebrates, which have been in existence for 700–800 million years [4]. They house a dense and diverse microbial population, which is believed to contribute to their unmatched prolific production of bioactive metabolites [3]. These bioactives are thought to play an important protective role against pathogens which might enter the sponge habitat. *Bacillus* species appear to be part of the microbiota of many marine sponges [5,6,7,8], although their diversity, properties and specific ecological contribution still remain poorly understood [9]. 

*Bacillus* are renowned for their ability to produce a wide variety of bioactive compounds [10,11]. The production of antimicrobials coupled with the development of environmentally highly resistant endospores, facilitates their survival in competitive ecological niches such as that which they are likely to encounter within sponges. *B. subtilis* is the best characterised member of the genus. Although initially described as a soil dwelling bacterium, numerous reports suggest that this species can be isolated from, and can grow, sporulate and germinate in, many different ecological niches [9,12,13,14,15,16,17]. *B. subtilis* isolates produce a vast array of chemically different antimicrobial peptides, which include non-ribosomally synthesised peptides, such as polymyxins and the lipopetide surfactin, gene encoded bacteriocins, such as subtilosin and, within this latter group, lantibiotics, such as subtilin [10]. 

Lantibiotics are ribosomally synthesised post-translationally modified antimicrobial peptides typically produced by Gram-positive bacteria, although the array of producer organisms is currently expanding to other groups of bacteria [18]. Lantipeptides, *i.e.*; the name used to describe lantibiotics and related peptides which lack antimicrobial activity, are currently classified into four classes (I to IV) according to the biosynthetic pathway involved in their production. While dedicated dehydratase (LanB) and cyclase enzymes (LanC) are involved in the modification of class I lantibiotics, for the other three classes this is mediated by a single multifunctional enzyme [18,19]. 

The bio-conversion from a linear lantibiotic precursor, with an *N*-terminal leader peptide and a *C*-terminal core peptide, to the mature active molecule involves different post-translational modification events of the *C*-terminal portion, such as the enzymatic conversion of serine and threonine residues into the dehydrated amino acids dehydroalanine (Dha) and dehydroamino-2-butyric acid (Dhb), respectively. When one of these modified amino acids interacts with an intrapeptide cysteine, a thioether bond is formed generating *meso*-lanthionine (Lan) or (2*S*,3*S*,6*R*)-3-methyllanthionine (MeLan) [20]. Besides these, other less common modifications have also been described [19]. The post-translational modifications appear to influence not only the structure of the peptides, but also their activity and stability against protease degradation and heat denaturation [18,21,22]. For class I lantibiotics, the mature peptide is then translocated through the membrane generally by a transmembrane ATP-binding cassette (ABC) transporter (LanT). This can happen before or after cleavage of the leader peptide from the modified core peptide, which is frequently mediated by a dedicated serine protease LanP [19]. Dedicated immunity proteins (LanI) and/or specialised ABC transporter proteins (LanFE(G)) protect the host from the action of its own lantibiotic. All of the genes involved in the processing, transport, immunity and regulation are organised in genetic clusters, which are frequently composed of multiple transcriptional units. While the mode of action of most lantibiotics is thought to involve inhibition of peptidoglycan biosynthesis through their interaction with specific molecular targets, such as the cell wall precursor lipid II, many will additionally cause pore formation in the cytoplasmic membrane and ultimately, result in cell death [19].

*Bacillus* lantibiotics, despite their broad spectrum of antimicrobial activity, have attracted limited attention regarding their potential applicability in food, agricultural and clinical fields. Endospore-forming bacteria, particularly *Bacillus*, are known to play an important role in the preparation of Asian fermented foods [23,24] and are currently being used as spore probiotic preparations in human therapy, animal production and aquaculture [14,25,26]. In this regard, the production of antimicrobial compounds, such as lantibiotics, is regarded as a highly desirable property if candidate strains are to be used as probiotics and/or starter cultures. 

Despite several reports describing the isolation of bioactive *Bacillus* strains from marine sponges [27,28,29], very few have successfully identified the associated antimicrobial compounds. In this study we identify and characterise a novel class I lantibiotic, here named subtilomycin, produced by *B. subtilis* MMA7 isolated from the marine sponge *Haliclona simulans*. 

## 2. Results

### 2.1. Antimicrobial Activity of *B. subtilis* Strain MMA7

Strain *B. subtilis* MMA7 isolated from the marine sponge *H. simulans*, was previously shown to display a strong antimicrobial activity against different indicator bacteria [9]. The spectrum of activity of this isolate has been extended in the current study to include marine associated Gram-negative bacteria, such as *Aeromonas hydrophila*, *Vibrio anguillarum* and *Alteromonas* sp., and nosocomial pathogens, such as VRE, VISA, hVISA and MRSA (Table 1 and Table S1). Strong inhibitory activity was also seen towards multiple *Candida* species, such as *C. albicans*, *C. dubliniensis*, *C.* *lusitaniae* and *C. parapsilosis*.

**Table 1:** Antimicrobial activity of *B. subtilis* strain MMA7 and derived bioactive peptide ^#^.

Indicators	MMA7 culture	Peptide
*B. cereus*	+++	++
*B. megaterium*	++++	++
*L. monocytogenes*	++++	++
*L. innocua*	++++	++
*C. sporogenes*	+++++	+++
*C. perfringens*	++++	- *
*C. difficile*	++	- *
*E. faecium*	++	- *
*S. aureus*	++	+
MRSA	++	+
hVISA	+	+
VRE	+	- *
*L. lactis HP*	++++	+
*A. hydrophila*	++++	+ *
*V. anguillarum*	+	+ *
*Alteromonas* sp.	+++	+ *
*P. aeruginosa*	-	+ *
*C. albicans*	++++	- *
*C. dubliniensis*	++	- *
*C. lusitaniae*	++	- *
*C. parapsilosis*	+	- *

^#^ Producer *B. subtilis* strain MMA7 was grown on Marine agar (MA) for the deferred antagonism assays. Antimicrobial activity of purified peptide (~10 μM) derived from the MMA7 *∆sbo-albF::cat* mutant strain was tested by a spot on lawn assay. Testing was repeated at least three times and representative results are shown; +, clear halo of growth inhibition; multiple +, increased activity as assessed visually by the increased diameter of the inhibition zone; -, no inhibition; * Results detected with 100 fold more peptide than that used for the susceptible Gram-positive indicators. No activity was detected on deferred antagonism assays against *Escherichia coli*, *Enterobacter aerogenes*, *Salmonella typhimurium*, *Burkholderia cenocepacia*, *Stenotrophomonas* *maltophilia*, *Klebsiella pneumoniae*, *Serratia marcescens*, *Morganella morganii* and *Candida glabrata*.

**Figure 1:**
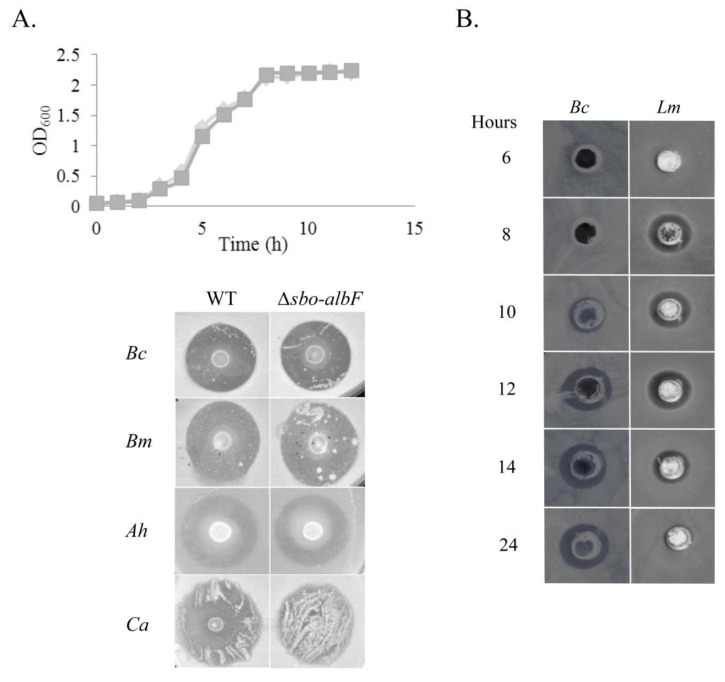
Antimicrobial activity of *B. subtilis* strain MMA7. (**A**) Growth of the wild type (WT) strain *B. subtilis* MMA7 (diamond) and the *∆sbo-albF::cat* mutant strain (squares) in MB (top panel). Antimicrobial activity of the WT and the ∆*sbo-albF::cat* mutant strains, tested on a deferred antagonism assay against *B. cereus* (*Bc*), *B. megaterium* (*Bm*), *A. hydrophila* (*Ah*) and *C. albicans* (*Ca*) (bottom panel); (**B**) Kinetics of production of antimicrobial compounds by *B. subtilis* strain MMA7. Antimicrobial activity of concentrated cell-free supernatants from samples collected at different time points of the bacterial growth was tested on a well diffusion assay against the indicators *B. cereus* (*Bc*) and *L. monocytogenes* (*Lm*). All experiments were repeated twice and representative results are shown.

Antimicrobial activity by *B. subtilis* strains is commonly associated with the production of bacteriocins. In order to test for the presence of genes encoding the *B. subtilis* bacteriocins subtilosin, sublancin or subtilin in strain MMA7, PCR primers sets were designed to amplify sequences corresponding to the associated biosynthetic gene clusters (Table S2). While no amplification was observed with primers targeting sublancin and subtilin, a PCR product was obtained with subtilosin-specific primers. However, no marked difference in antimicrobial activity was observed between a deletion insertion mutant of the subtilosin biosynthetic cluster (*∆sbo-albF::cat*) of *B. subtilis* MMA7 and the wild type strain (Figure 1A). This suggested the likely production of other antimicrobial compound(s) by the strain. Although all the work hereafter was carried out on the mutant background, for simplicity it will be referred to as strain MMA7.

#### Kinetics of Antimicrobial Activity

In order to establish the kinetics of production of the bio-active compound(s), the producing strain was grown in Marine Broth (MB) and the bioactivity of freeze-dried cell free supernatants collected at the different time points examined on a well assay against different indicator strains. Inhibitory activity was initially detected at early stationary phase (approx. 8–10 h post-incubation) (Figure 1B). However, there was a clear difference between the spectrum of activity of MB supernatants collected after 12 h incubation *versus* those collected after 24 h (Figure 1B). While 12 h and 24 h concentrated supernatants showed identical activity against some of the indicators, such as *B. cereus*, the activity detected with 12 h supernatants against *L. monocytogenes*, was not evident from 24 h samples. The latter were also inactive against the indicators *C. sporogenes* and *L. lactis* (data not shown). 

### 2.2. Purification and Characterisation of the Antimicrobial Compound from 12 h Cultures

**Figure 2:**
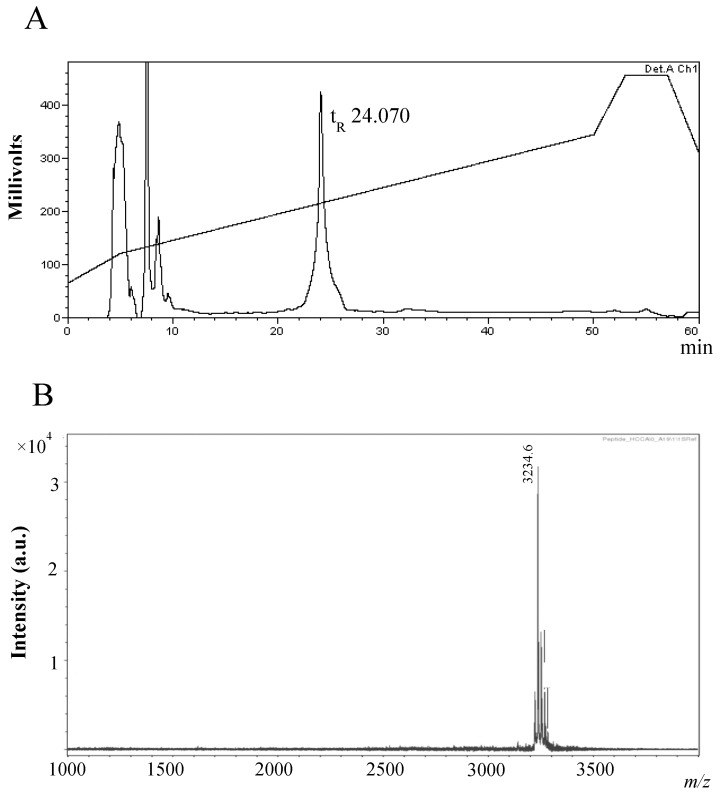
Purification of the *B. subtilis* strain MMA7 antimicrobial compound. (**A**) RP-HPLC purification of the antimicrobial compound present in ammonium sulphate crude extracts (ASCE) from 12 h MB cultures. A single peak was eluted with an acetonitrile gradient after the injection of 1 mL ASCE (retention time, 24.070 min/46% acetonitrile); (**B**) Mass spectrometry analysis of the RP-HPLC purified sample showing a single compound with a low molecular mass.

The purification strategy for the antimicrobial compound associated with the activity detected from 12 h MB supernatants, involved an initial ammonium sulphate precipitation step (40% saturation). Aliquots of the active ammonium sulphate crude extract (ASCE) were subsequently purified by Reverse Phase High Performance Liquid Chromatography (RP-HPLC) on a C12 column, and a single peak with a retention time (*t*_R_) of 24.070 min, at approximately 46%, on the acetonitrile gradient, was observed (Figure 2A). 

MALDI TOF MS analysis of the active RP-HPLC fractions revealed a singular molecular mass of 3235 Da (Figure 2B). Searches on different antimicrobial databases returned no match for the proposed mass. Hydrolysis of the compound purified by RP-HPLC and subsequent amino acid detection confirmed the peptide nature of the compound (data not shown).

The activity of the purified compound (~10 μM) was then tested against all the strains which were initially used as indicators on the deferred antagonism assay with the producing strain MMA7 (Table 1). While inhibitory activity was readily detected against most of the Gram-positive indicators, the strong activity seen on the deferred antagonism assay against *V. anguillarum*, *A. hydrophila* and *Alteromonas* sp., although clear, was only detected with 100 fold more compound than that required to inhibit the Gram-positive indicators. 

#### Physicochemical Characterisation of the Antimicrobial Peptide

Dried pellets originated from 1.5 mL RP-HPLC-purified peptide containing fractions were fully soluble in water, and aliquots of these samples retained activity against *L. monocytogenes* and *B. cereus* on a spot on lawn assay when stored at −20 °C and −80 °C for a period of up to four months. No activity was however detected after five months storage. Identical samples stored at 4 °C and room temperature lost their activity after two weeks.

**Figure 3:**
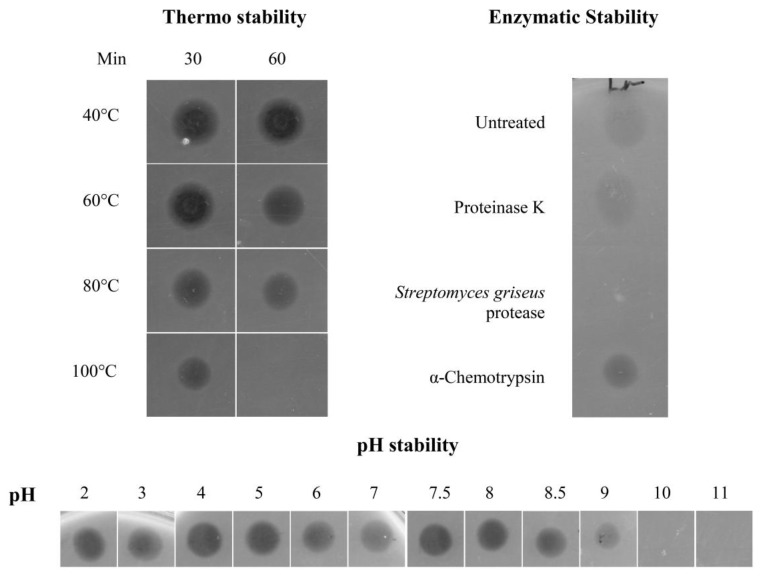
Thermal, pH, and proteolytic stability of the purified peptide. Activity of the bioactive peptide samples (~10 μM) treated in the different ways was assessed by a spot on lawn assay. Five μL of treated/control samples were spotted onto BHI agar plates seeded with *L. monocytogenes* to an OD_600_ of 0.015. Control samples containing the different proteolytic enzymes and no peptide, had no detectable inhibitory effect on the indicator strain. All assays were repeated at least three times, and representative results are shown.

The activity of the peptide was stable over a wide range of temperatures, including 100 °C for 30 min (Figure 3). The purified peptide was also fully active over a wide pH range (2 to 8.5), and while some activity was still seen at pH 9, no activity was detected at higher pH values (Figure 3). Susceptibility to proteolytic degradation was established by treating the purified peptide with proteinase K, α-chymotrypsin and a protease from *Streptomyces griseus*. None of the treatments resulted in a decrease of activity of the antimicrobial peptide, with the exception of a complete loss of activity after treatment with the *Streptomyces griseus* protease (Figure 3).

### 2.3. Genetic and Structural Characterisation of the Antimicrobial Peptide

#### 2.3.1. Primary Amino Acid Sequence of the Antimicrobial Peptide

MS and MS/MS analysis showed that the intact mass of the peptide has a number of oxidised forms (Figure S1), typical of the presence of thioethers, indicating that the compound was potentially a lantibiotic. 

Initial structural analysis of the peptide proved problematic given that Edman degradation was not possible as the *N*-terminal end of the peptide was blocked. Trypsin digestion and alkylation of the peptide followed by MALDI MS and MS/MS analysis however provided a tentative primary amino acid sequence of the *N*-terminal part of the peptide; and established that the peptide contained at least six or seven unusual amino acids which are common in lantibiotic peptides, such as Dhb, Dha and lanthionine.

The presumptive amino acid sequence was then used in subsequent BLASTp and TBLASTn searches against the draft genome sequence of *B. subtilis* MMA7. Following theses searches, homology was detected with a small protein-coding gene. This gene is located in the vicinity of protein-coding genes showing similarities to genes encoding lantibiotic modification enzymes. This allowed for the aforementioned experiments to be repeated, refined and the *N*-terminal sequence of the peptide to be confirmed as T-W-A-T-I-G-K-T-I-V-Q-S-V-K-K. We have here named this novel lantibiotic as subtilomycin.

#### 2.3.2. Genetic Organisation and Location of the Subtilomycin Biosynthetic Gene Cluster

**Figure 4:**
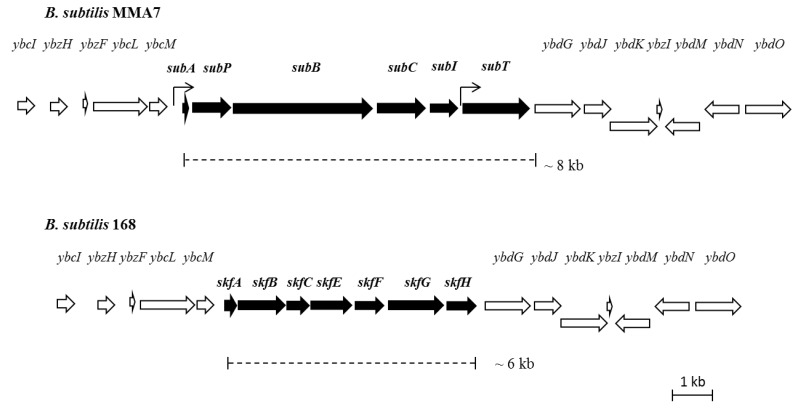
Structural organisation of the putative subtilomycin biosynthetic cluster and flanking regions: *subA*, subtilomycin structural gene; *subP*, serine protease; *subB*, lanthionine dehydratase; *subC*, lanthionine synthetase; *subT*, ABC transporter. The function of *sub*I cannot be predicted from its sequence, although its genetic location and lack of homologues is consistent with a possible involvement in immunity. Predicted promoters are indicated by arrows. A comparison of the genomic location of the subtilomycin biosynthetic cluster in strain MMA7 with that of the *skf* operon in strain 168 is provided.

As previously noted, a single match was found between the primary amino acid sequence of the *N*-terminal part of subtilomycin and the 5’end of a small gene (*subA*, 171 nt), located immediately upstream from ORFs that encode for proteins which have been shown to be involved in the processing of class I lantibiotics, such as LanB-like dehydratase and LanC-like cyclase modification enzymes (SubB and SubC, respectively, Figure 4). These three genes are clustered with two other ORFs that encode a putative extracellular serine protease (*subP*) and an ABC transporter (*subT*), respectively. These are likely to be involved in the processing and transport of the lantibiotic. The final ORF within the cluster (*subI*) has no homology to any other sequence in the database and could encode an immunity protein (Table S3). The ORFs flanking these sequences show high homology (greater than 95%) with the genome of *B. subtilis* 168, including a putative two-component response regulator, *ybdJ*, and sensor histidine kinase, *ybdK*, genes located downstream of the cluster (Figure 4). *In silico* analysis of the biosynthetic cluster identified two putative promoter sequences, located upstream of *subA* and *subT*, respectively.

Further sequence analysis of the flanking regions of the putative subtilomycin biosynthetic gene cluster allowed us to establish that the cluster is inserted in the same genomic locus as the sporulation killing factor (*skf*) operon in *B. subtilis* subsp. *subtilis* strain 168 [30] (Figure 4). The nucleotide sequence encompassing the putative subtilomycin biosynthetic cluster and neighbouring genes has been deposited in GenBank under accession number JX912247. 

#### 2.3.3. Further Structural Characterisation of Subtilomycin

The presence of an *N*-terminal 2-oxo butyrate residue was established by MS/MS analysis of the peptide. Such residues arise as a consequence of the fact that *N*-terminal Dhb residues are not stable and undergo spontaneous oxidative deamination [31]. The deaminated Dhb (Dhb*) weighs 85 Da instead of 83 Da, resulting in a mass gain of 2 Da.

The mass difference from the alanine at position 3 to the *N*-terminal of subtilomycin equals 271.3 Da, which corresponds to the sum of 186.07 Da for the tryptophan residue with 85.03 Da for the deaminated Dhb. The *C*-terminal of the peptide was not suitable for *de novo* sequencing, but data from the analysis of tryptic digests suggested that this region of the peptide could possibly contain up to five ring structures, three Lan and two MeLan, since five cysteines, three Dha and two Dhb are present in this section of the peptide.

Therefore the most likely sequence for the lantibiotic is Dhb*-W-A-Dhb-I-G-K-Dhb-I-V-Q-Dha-V-K-K-C-R-Dhb-F-Dhb-C-G-C-Dha-L-G-Dha-C-Dha-N-C-N, where the underlined amino acids have been experimentally confirmed by mass spectrometric analysis and the subsequent sequence predicted from the genomic sequence of strain MMA7. There was no mass change following DTT reductions suggesting that no disulphide bonds were present in the peptide. In summary, the 32 amino acid long lantibiotic contains nine modified aminoacids, five of which are likely to be involved in the formation of thioether bridges. The theoretical mass of the proposed sequence is 3234.5, while the observed mass was 3234.6 (*m*/*z* = 3235.6). 

**Figure 5:**
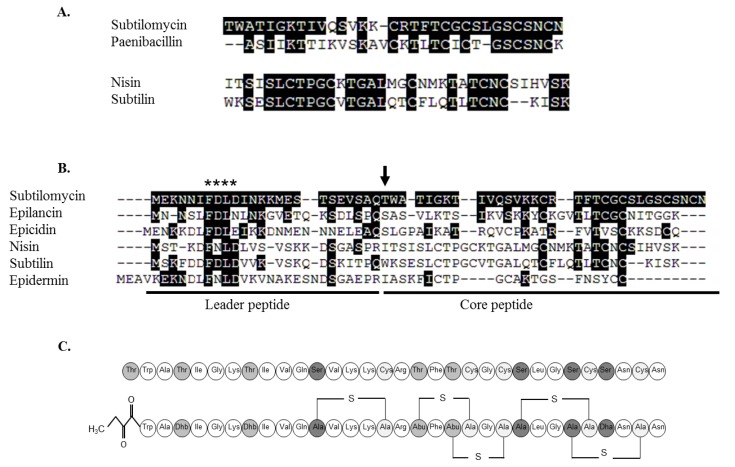
Comparison of the amino acid sequence between subtilomycin and nisin pro-petides and their respective closest homologue (**A**). Comparison of the amino acid sequence of the *N*-terminal leader peptide and the *C*-terminal core peptide of subtilomycin and different class I lantibiotics (**B**). Sequences obtained from Uniprot were aligned with ClustalX (Multiple Sequence Alignment version 2.0.11, [34]) and sequence analysis processed with GeneDoc (Multiple sequence Alignment Editor & Shading Utility, Version 2.7.000). Conserved amino acids are boxed in black and gaps are indicated by hyphen. A vertical arrow indicates the first amino acid of the pro-peptide *, indicates the conserved motif of class I leader peptides documented to be important for efficient production. Proposed conformational structure of the mature lantibiotic subtilomycin (**C**). Top, unmodified propeptide. Bottom, mature peptide, where Ser and Thr residues which are posttranslationally dehydrated to Dha and Dhb, or involved in the formation of Lan and MeLan, respectively, with cysteine residues, are shaded in grey. The location of the thioether bridges was estimated from the amino acid sequence and by comparison with that of paenibacillin [32]. The presence of the *N*-terminal 2-oxobutyrate residue is also indicated.

Search of the bactibase and the antimicrobial peptide databases highlighted some similarities with previously identified lantibiotics. The closest homology was with paenibacillin produced by *Paenibacillus polymyxa* OSY-DF (54%) [32,33], followed by epilancin 15X produced by *Staphylococcus epidermidis* 15X154 (43%) (Figure 5A,B). Although the structure of purified paenibacillin has been elucidated, the genetics of the associated biosynthetic cluster have not been published. As a result comparative analysis of the paenibacillin and subtilomycin pre-peptides (Figure 5B) and respective gene clusters was not possible. 

Based on the primary amino acid sequence of subtilomycin, we have here proposed a structure for subtilomycin (Figure 5C), which reflects that experimentally established for paenibacillin [32]. However further analysis will be required to definitively establish the proposed structure. 

### 2.4. Distribution of the Subtilomycin Biosynthetic Gene Cluster

**Figure 6:**
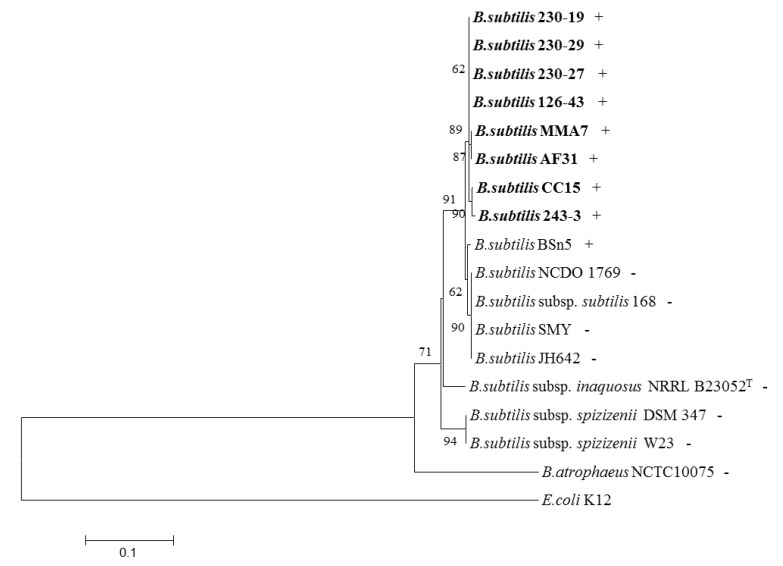
Neighbour joining phylogenetic tree of *gyrA* gene sequences from different *B. subtilis* strains. Coastal (MMA7, CC15, AF31) and deep water (230-19, 230-29, 230-27, 126-43, 243-3) marine sponge-associated isolates are highlighted in bold. + and -, after strain designation, indicates the presence and absence of the subtilomycin structural gene, *subA*.

Different secondary metabolites, and bacteriocins in particular, appear to have distinct distribution or prevalence patterns among related bacteria. A group of *B. subtilis* strains isolated from different coastal and deep water marine sponges (Table S1) were thus screened with PCR primers targeting the subtilomycin structural gene, *subA* (Table S2). A PCR product was detected in all of the sponge-derived strains tested, but was absent in other non-marine derived *B. subtilis* strains which were screened either by PCR or by in silico analysis of their genome (Figure 6). The only exception in the latter group was the recently sequenced endophytic *B. subtilis* strain BSn5, isolated from *Amorphophallus konjac* [35].

In order to establish the phylogenetic relationship between the sponge isolates with their counterparts, the *gyrA* gene sequences obtained from the strains, using *gyrA* specific PCR primers (Table S2) were used to generate a Neighbour-joining phylogenetic tree (Figure 6). This analysis indicated that the sponge isolates although, more closely related to *B. subtilis* subsp. *subtilis* than to the other subspecies, appear to form subgroups which cluster together, and are more closely related to each other than to other, non-sponge, isolates. 

## 3. Discussion

During our search for new antimicrobial compounds produced by marine sponge associated bacteria, *B. subtilis* strain MMA7 isolated from *H. simulans* demonstrated broad range antimicrobial activity towards important clinical and aquaculture pathogens. Interestingly, activity against a number of indicator strains, such as *L. monocytogenes* and *C. sporogenes*, was detected from 12 to 14 h cultures but not from 24 h cultures. This activity was here shown to be associated with a novel lantibiotic, subtilomycin. Lantibiotics are typically produced by specific strains and in general target Gram-positive organism, although examples are emerging of those that have an extended spectrum of activity that includes Gram-negative bacteria [18,36]. Subtilomycin shows antimicrobial activity against a broad spectrum of Gram-positive and some Gram-negative bacteria, although the susceptibility of the latter group appears to be significantly less than the former. It also possesses several physicochemical properties which make it interesting for use in the food and/or pharmaceutical industry, including solubility in water and stability under different conditions such as physiologic pH. 

Based on MS, MS/MS and genome sequence analysis the subtilomycin precursor undergoes extensive posttranslational modification. Three Dhb residues are present at positions T1, T4 and T8. However, similar to Pep5 and Lacticin 3147A2, the *N*-terminal Dhb is modified to 2-oxo butyrate [31,37]. The post translational modification of the *N*-terminal residues in lantibiotics is believed to provide protection from aminopeptidases [18]. The remaining threonine residues, T18 and T20, are involved in the formation of β-methyllanthionines. Additionally, one Dha residue, and three lanthionines result from serine residues at positions 12, 24, 27 and 29, respectively. The closest homologue to subtilomycin is paenibacillin, a 30 amino acid long lantibiotic (*m*/*z* = 2984.61) isolated from the sporeformer *P. polymyxa* OSY-DF [32]. The two molecules seem to be particularly similar at the *C*-terminal region with a similar ring pattern structure, a feature which is also seen between nisin and its closest relative subtilin [38]. This could be associated with the similarities found within the physicochemical properties described for both subtilomycin and paenibacillin [32,33]. However, in contrast paenibacillin has an *N*-terminal acetylation rather than 2-oxo butyrate. Also, although subtilomycin could be detected from 12 h MB cultures but not from 24 h fermentates, paenibacillin could be equally detected from both time points [32], which may indicate differences in the regulation of production. 

The proposed subtilomycin biosynthetic gene cluster appears to have several of the sequence features associated with class I lantibiotics [39], including the presence of LanP, LanB and LanC-like encoding sequences immediately downstream from the structural gene *subA*. The conserved four amino acid residue box (FNLD) of class I leader peptides known to be important for efficient production [18] is also present in the leader peptide of subtilomycin supporting its grouping with class I lantibiotics. Contrary to the other aforementioned elements involved in the synthesis of lantibiotics, there is very little homology between the peptides involved in providing immunity to the different lantibiotics [19]. With this in mind, we propose that SubI, which has no homologues on the database, could be fulfilling this role. If this is confirmed, similarly to Pep5 and lactocin S, host resistance to subtilomycin, would be provided by a single dedicated immunity protein, dispensing the additional ABC transport proteins. These questions will be the focus of further investigations. 

The subtilomycin biosynthetic cluster in *B. subtilis* strain MMA7 is found in place of the *skf* operon, which is present in different *B. subtilis* isolates, such as strain 168. This encodes for production, export and immunity towards the sporulation killing factor (SkfA). This peptide mediates a well characterised bacterial cannibalistic behaviour intended to delay sporulation, by which producing sporulating sibling cells feed on non-producing non-sporulating cells by mediating their lysis [30]. It will be interesting to establish if the lack of the *skf* operon in strain MMA7 results in any change in the temporal entry to sporulation. Of note, the rhizocticin biosynthetic gene cluster (*rhi*) of *B. subtilis* ATCC6633 was also inserted nearby, and in place of, the *skf* operon [40]. This may indicate that in the *B. subtilis* genome this may be a hotspot for the location of genetic elements associated with the production of secondary metabolites. It will be interesting to survey different natural *B. subtilis* isolates at this site to see the prevalence and identity of these elements. Importantly, the GC content of the proposed subtilomycin biosynthetic gene cluster (29.5%) is considerably different to the overall GC content of the genome of strain MMA7 (44%), suggesting a potential exogenous acquisition of this cluster. 

The structural gene coding for subtilomycin has been found in several other *B. subtilis* strains isolated from different shallow and deep sea marine sponges, but appears to be absent from many *B. subtilis* isolates from other environments, with the exception of strain BSn5, an endophytic *B. subtilis* from *Amorphophallus konjac* [35]. Interestingly, a closer phylogenetic relationship based on analysis of *gyrA* sequences, exists between the marine isolates, than with the other strains. This closer phylogenetic relationship occurs despite clear morphological and phenotypic differences between the marine sponge isolates (data not shown). Apart from its industrial and pharmaceutical potential the biological role of subtilomycin remains unknown, although its presence in all the marine sponge *B. subtilis* isolates may indicate a potential niche-related role associated with providing the strain with some competitive advantage within the stringent environment found within marine sponges. Nevertheless, its presence in the endophytic BSn5 isolate may indicate a more complex role in the symbiotic relationship with the host. 

## 4. Experimental Section

### 4.1. Bacterial Strains, Media and Growth Conditions

Bacterial strains used in this study are listed in Table S1. *B. subtilis* strain MMA7, was isolated from the marine sponge *H. simulans* collected during scuba diving in Gurraig Sound Kilkieran Bay, Galway, on the west coast of Ireland [9]. Strains AF31 and CC15 were isolated from the shallow water sponges *Amphilectus fucorum* and *Cliona celata*, respectively, both collected from the Lough Hyne Marine Reserve, Ireland, by Scuba diving. Strains BD230-19, BD230-27, BD230-29, BD243-3 and BD126-43 were isolated from the deep-water sponges BD230, BD126 and BD243, collected from the Rockall Ridge and Porcupine Bank, 300 nautical miles North West of Galway harbour, at a depth of 2900, 2129 and 1300 m, respectively. Unless stated otherwise, marine sponge *B. subtilis* isolates were routinely grown and maintained aerobically on Difco™ Marine agar (MA) and broth (MB) (Difco 2216), at 30 °C. Other strains used in this study, including indicator strains, were grown in the media specified in Table S1, aerobically at 37 °C, with the exception of *Aeromonas hydrophila*, *Vibrio anguillarum*, *Carnobacterium malteromaticum* and all the *Candida* strains that were incubated at 30 °C. *Clostridium perfringens* and *Clostridium sporogenes* were grown anaerobically in an anaerobic jar at 37 °C. *E. coli* DH5α used for cloning experiments was grown in Luria-Bertani (LB). For *B. subtilis* strains chloramphenicol was used at 5 μg·mL^−1^.

### 4.2. Antimicrobial Activity Screening Assays

Antimicrobial activity of producing strains against the indicator strains was initially assessed with a deferred antagonism assay as described by Phelan *et al.* [9]. Subsequent testing of freeze dried crude extracts and purified compounds was carried out with a well and/or a spot on lawn assays. Essentially, 5 μL of samples were spotted onto 20 mL agar plates seeded with the indicator strain to a final OD_600_ of 0.015, or 30 μL aliquots were applied to a 5 mm well made on identical plates. Controls of freeze dried MB or solvent were tested in parallel.

### 4.3. DNA Extraction, PCR Amplification, DNA Sequencing and Phylogenetic Analysis

Genomic DNA was extracted from 5 mL overnight MB cultures by a modification of the guanidine thiocyanate method described by Pitcher *et al.* [41]. Plasmid DNA was purified from 5 mL overnight *E. coli* LB cultures with the QIAprep spin Miniprep Kit (Qiagen, GmbH, Hilden, Germany). 

All oligonucleotide primers used in this study are listed in Table S2. Unless stated otherwise PCR reactions (50 μL) contained 1× BioTaq PCR buffer (Bioline, London, UK), 1.5 mM MgCl_2_, 0.2 mM dNTPs, 0.5 μM of each primer, 2.5 U BioTaq DNA polymerase (Bioline), and 3 μL bacterial lysates or 50 ng of genomic DNA as template. With the exception of the annealing temperature (Table S2), and the extension time, the cycling parameters for the amplification of the different target sequences remained constant. An initial denaturation at 94 °C for 4 min, was followed by 30 cycles of 94 °C for 30 s, X °C for 30 s, and 72 °C for X min, with a final extension of 10 min at 72 °C. When required, PCR products were purified using the QIAquick PCR purification kit (Qiagen GmbH, Hilden, Germany). Routine DNA sequencing was performed by GATC-Biotech, AG (Konstanz, Germany).

Partial *gyrA* gene sequences of the marine sponge *B. subtilis* isolates were deposited in GenBank with the following accession numbers: CC15, JX977124; AF31, JX977125; 243-3, JX977126; 230-19, JX977127; 230-29, JX977128; 126-43, JX977129; 230-27, JX977131; MMA7, JX977130. For phylogenetic analysis, 620 nt of the *gyrA* genes (nt 232 to 852 in the *E. coli* *gyrA* gene, accession number AP012306.1) were aligned using Clustal X2 [34]. Neighbour-joining phylogenetic trees were constructed using MEGA 4 [42] and bootstrap tests were performed once with 1000 replicates. 

### 4.4. Construction of Strain MMA7 ∆Sbo-albF::Cat Mutant

Methods for the preparation and transformation of *E. coli* DH5α and *B. subtilis* competent cells were as described previously [43,44]. To create a *sbo-albF* insertion-deletion mutant, DNA fragments containing 284 nt of the upstream and coding region of the structural gene, *sbo* (nt 136 to 420, accession number AJ430547) and 284 nt internal nucleotides of the *albF* gene (nt 5684 to 5968, accession number AJ430547), were PCR amplified from the genomic DNA of strain MMA7 with the primer pair SboUp-F/Sbo-R and AlbF-F/AlbF-R, respectively (Table S2). Digested amplified fragments were sequentially inserted between the *Hin*dIII-*Bam*HI sites and *Eco*RI-*Xho*I sites flanking the chloramphenicol resistance cassette in the pMK3 derivative integrational vector, pMS38 [45]. The resulting construct was named pRP1. The mutant strain, MMA7Δ*sbo-albF*::*cat*, Chl^R^, in which the *sbo-albF* region (nucleotides 420 to 5684, accession number AJ430547) was replaced by a chloramphenicol cassette by a double-crossover event, was generated by the transformation of strain MMA7 with *Sca*I linearised pRP1, with subsequent selection for chloramphenicol resistance (Chl^R^). Disruption of the cluster in the mutants was confirmed by PCR with a combination of primers specific for the chloramphenicol cassette, and primers flanking or internal to, the deleted region. Specifically, primer pairs ywiB-F & albA-R2, ywiB-F & cat255-R, albD-F & alb*-*FDown-R, cat958-D & albFDown-R (Table S2). 

### 4.5. Kinetics of Antimicrobial Production

MB (100 mL) were inoculated with overnight MB cultures of the producing strain (1:100) and grown at 30 °C, 150 rpm shaking on an orbital incubator. The optical density (600 nm) was read hourly until 14 h incubation and then at 24 h incubation and 5 mL aliquots removed at each time point and centrifuged at 3200 rpm, 15 min, 20 °C. Supernatants were filter sterilised (33 mm Cellulose Acetate, 0.45 μm syringe filters, Anachem Ltd., Luton, UK), and freeze dried (Labconco Freezone6, Labconco Kansas City, MO, USA) for 16 h. Freeze dried material was resuspended in 500 μL of sterile HPLC grade water and tested for activity in a well diffusion assay against different indicator strains.

### 4.6. Purification and Characterisation of the Antimicrobial Compound

Approximately 1 L of *B. subtilis* MMA7 12 h MB cultures were centrifuged at 3200 rpm, for 15 min at 20 °C and the supernatant filter sterilized using a Nalgene^®^ vacuum filtration system (0.45 μm, Sigma-Aldrich^®^, Munich, Germany). Ammonium sulphate was added intermittently to the cell free supernatant to 40% saturation, and gently stirred overnight at 4 °C. The resulting precipitate was collected by centrifugation of 100 mL aliquots at 12,500 rpm, for 90 min, at 4 °C and resuspended in total volume of 2 mL of sterile HPLC grade water (Sigma-Aldrich, CHROMASOLV^®^ Plus, Munich, Germany) (1/500 of original volume). Subsequent purification by RP-HPLC was achieved by applying 1 mL of the ammonium sulphate crude extract onto a Phenomenex^®^ C12 column (Jupiter 4 μ proteo 90 Å, 250 × 10.0, 4 μm). Separation was carried out with a gradient of acetonitrile (33% to 65%) containing 0.1% TFA from 5 to 50 min, at a flow rate of 2.1 mL·min^−1^ and separation monitored at 214 nm. Fractions (1.5 mL) were evaporated to dryness on a speedvac (Savant DNA 120, Speedvac, Thermo Scientific, London, UK), and the resulting pellets resuspended in sterile HPLC grade water. The aforementioned purification process routinely yielded 0.4–0.5 mg of compound per litre of a 12 h culture.

The purity and molecular weight of the compound in the active fraction was initially estimated on an AXIMA-TOF^2^ MALDI Mass spectrometer (Shimadzu Biotech, Manchester, UK). Further analysis of the nature and structure of the compound was carried out by Alphalyse A/S (Odense, Denmark). This involved determination of the amino acid composition, MALDI MS and MALDI MS/MS analysis. 1.77 μg of RP-HPLC purified sample resuspended in 0.1% TFA was used for amino acid analysis. The acid hydrolysis was performed for 20 h at 110 °C, in 6 N HCl, 0.1% phenol, 0.1% thioglycolic acid. The hydrolysis took place under reduced pressure in an atmosphere of argon. Identification and quantification of the amino acids took place on a BioChrom 30 amino acid analyzer using ion exchange chromatography, post-column derivatization with ninhydrin and detection at two wavelengths, 570 nm and 440 nm. A known amount of the non-natural amino acid norleucine (Nle) was added as an internal control standard. 

For MALDI MS and MS/MS of intact peptide, approx. 5 pmol peptide was spotted onto a steel target with alpha-cyano-4-hydroxycinnamic acid (CHCA) matrix and was analyzed on a Bruker Autoflex Speed instrument. For MALDI MS and MS/MS analysis of trypsin digests, the peptide was digested with trypsin at 37 °C overnight and the resulting peptides were concentrated on a C18 ziptip from Millipore and eluted with CHCA matrix in 50% acetonitrile/0.1% TFA. Subsequent analysis involved alkylation of the peptide with β-mercaptoethanol in a basic solution. Alkylation increased the peptide mass by 78 Da for each alkylated amino acid. Samples were taken out at different times to follow the alkylation process. When samples were removed they were acidified with TFA and purified on a C18 ziptip from Millipore before being eluted with CHCA matrix in 50% acetonitrile/0.1% TFA and analysed by MALDI MS and MS/MS.

Reduction of the peptide with DTT was used to ascertain the potential presence of disulfide bonds, as the peptide mass would be increased by 2 Da when a disulfide bond is broken due to the addition of two hydrogen atoms.

### 4.7. Heat, pH and Proteolytic Treatment of the Antimicrobial Peptide

To establish the thermal stability of the purified peptide, aliquots (~10 μM) were exposed to 40, 60, 80 and 100 °C for 30 and 60 min respectively before being tested for bioactivity on a spot on lawn assay. Untreated samples were used as controls. In a similar way, samples were checked for activity following storage for different time periods at room temperature, 4 °C, −20 °C and −80 °C. 

The activity of the purified peptide at different pH values was established by resuspending the peptide in 50 μL of 100 mM Tris buffer (~10 μM), with pH values between 2 and 11. Samples were incubated for 1 h at room temperature before being tested for antimicrobial activity against *L. monocytogenes* on spot on lawn assays. The different buffers were also individually tested as negative controls.

Susceptibility to proteolytic enzymes was tested by incubating the purified peptide resuspended in water (~10 μM) with proteinase K, α-chymotrypsin and a protease mixture from *Streptomyces griseus* (Sigma Aldrich^®^ 81748), to a final concentration of 1 mg·mL^−1^, at 37 °C for 1 h. Samples were then heated to 100 °C for 2 min to inactivate the proteolytic enzymes and bioactivity was tested with a spot on lawn assay. 

### 4.8. Genome Sequencing of *B. subtilis* MMA7 and Sequence Analysis of the Subtilomycin Biosynthetic Gene Cluster

The genome of isolate MMA7 was sequenced by Genospec, Inc., (Houston, TX, USA) using Illumina Sequencing technology on a Illumina High Seq 2000 (Illumina Inc., California, CA, USA). The sequencing data, which provided 100X coverage of the genome, was assembled into 48 contigs, spanning approximately 4.2 Mb. A total of 4277 open reading frames (ORFs) were determined and annotated using the RAST Server (Rapid Annotation using Subsystem Technology) and the Basic Local Alignment Search Tool (BLAST) programme at NCBI [46]. Further details on the analysis of the genomic sequence of strain MMA7 will be published elsewhere.

BPROM and the Berkeley Drosophila Genome Project (BDGP) [47,48], promoter prediction algorithms were used to identify putative promoters within the subtilomycin gene cluster. Only promoters identified by both algorithms were given further consideration.

For comparison of the amino acid sequence of lantibiotic precursors sequences obtained from Uniprot, were aligned with ClustalX (Multiple Sequence Alignment version 2.0.11, [34]) and sequence analysis processed with GeneDoc (Multiple sequence Alignment Editor & Shading Utility, Version 2.7.000).

## 5. Conclusions

This study describes the identification of a novel class I lantibiotic, subtilomycin, produced by a marine sponge *B. subtilis* isolate. Lantibiotics are an important class of antimicrobial peptides with potential commercial application. Of relevance, subtilomycin possesses several physicochemical properties which make it interesting for use in the food and/or pharmaceutical industry.

Despite the abundance and broad range of mechanism of antimicrobial compounds produced by the marine sponge microbiota, to the best of our knowledge, subtilomycin is the first lantibiotic to be purified and identified from a marine sponge associated bacteria. This emphasises the importance of pursuing the search for novel antimicrobial compounds within this still untapped environment. It additionally reinforces the importance of the *Bacillus* genus as a prolific resource of new bioactive compounds.

## Supplementary Files

Supplementary File 1 Supplementary Information (PDF, 216 KB)
