# Supplementary material for: Subtilomycin: A New Lantibiotic from Bacillus subtilis Strain MMA7 Isolated from the Marine Sponge Haliclona simulans
Source: Mar Drugs. 2013 Jun 3;11(6):1878–98. doi: 10.3390/md11061878 (PMC3721211; doi:10.3390/md11061878)
Supplement: Supplementary File 1 — Supplementary Information (PDF, 216 KB) [file marinedrugs-11-01878-s001.pdf]

Supplementary Information

Table S1. Bacterial strains used in this study.

Bacterial strains	Growth media [a]/Origin [b]
Marine sponge *Bacillus subtilis*: BD126-43	MA, MB/This work
BD230-19	MA, MB/This work
BD230-27	MA, MB/This work
BD230-29	MA, MB/This work
BD243-3	MA, MB/This work
CC15	MA, MB/This work
AF31	MA, MB/This work
MMA7	MA, MB/[1]
Bacillus subtilis NCDO 1769	LB/MDCC UCC
Bacillus subtilis NRRL B-23052^{T}	LB/BGSC
Bacillus subtilis 168	LB/BGSC
Bacillus subtilis DSM 347	LB/DSMZ
Bacillus cereus NCIMB 9373	LB/MDCC UCC
Bacillus megaterium ATCC 19213 (BGSC 7A2)	LB/BGSC
Staphylococcus aureus NCDO 949	BHI/MDCC UCC
MRSA ST 530	BHI/MDCC UCC
VISA22784	BHI/MDCC UCC
hVISA 35197	BHI/MDCC UCC
VRE EC725	BHI/MDCC UCC
Enterococcous faecium NCIMB 11508	BHI/MDCC UCC
Listeria innocua DPC 3567	BHI/MDCC UCC
Listeria monocytogenes EGDe	BHI/MDCCUCC
Clostridium perfringens NCDO 1799	TGB/MDCC UCC
Clostridium sporogenes NCDO 1791	TGB/MDCC UCC
Clostridium difficile 001	BHI/MDCC UCC
Carnobacterium malteromaticum LMG 9839	TSB/BCCM™/LMG BC
Lactococcus lactis subsp. *cremoris* HP	GM17/MDCC UCC
Escherichia coli NCIMB 15943	LB/MDCC UCC
Escherichia coli MUH 103	LB/[2]
Enterobacter aerogenes NCIMB 10102	LB/MDCC UCC
Salmonella Typhimurium LT2	LB/MDCC UCC
Pseudomonas aeruginosa PAO1	LB/MDCC UCC
Burkholderia cenocepacia J2315	LB/[3]
Stenotrophomonas maltophilia K279A	LB/[4]
Klebsiella pneumoniae MUH 588	LB/[2]
Serratia marcescens MUH 436	LB/[2]
Morganella morganii MUH 988	LB/[2]
Aeromonas hydrophila LMG 2844	TSB(NA)/BCCM™/LMG BC
Listonella (*Vibrio*) *anguillarum* LMG 4410	MB/DSMZ
Candida glabrata	YPD/MDCC UCC
Candida albicans SC5314	YPD/MDCC UCC
Candida dubliniensis	YPD/MDCC UCC
Candida lusitaniae	YPD/MDCC UCC
Candida parapsilosis	YPD/MDCC UCC

[a] Standard media were: LB, Luria Bertani; TGB, Thioglycolate broth (Merck); YPD, Yeast extract peptone dextrose media; BHI, Brain heart infusion (Merck); MA, Marine Agar; MB, Marine broth (Difco); TSB, Tryptic soya broth (Merck); NA, Nutrient agar (Difco); GM17, M17 broth (Merck) supplemented with

Glucose 0.5%; [b] Bacterial strains were obtained from the Microbiology Department Culture Collection, University College Cork (MDCC UCC), the *Bacillus* Genetic Stock Centre (BGSC), Belgium Coordinated Collections of Microorganisms (BCCM™)/LGM Bacteria Collection and DSMZ, DSM Collection, Deutsche Sammlung von Mikroorganismen und Zellkulturen GmbH, Braunschweig, Germany. MRSA, methicillin resistant *Staphylococcus aureus*; VISA, vancomycin intermediate *Staphylococcus aureus*; *hVISA*, heterogeneous VISA, and VRE, vancomycin resistant enterococci.

Table S2. Oligonucleotide primers used in this study.

**Primer**	**Sequence (5′-3′) ***	**Annealing (℃)**
Construction of MMA7 Δ*sbo-albF::cat* mutant		
sboUp-F (HindIII)	CCCAAGCTTCCATCATTGCTCATCAGATTTGA	58
sbo-R (BamHI)	CGCGGATCCTCCGATCGAGCATGTTGCACAA	
albF-F (EcoRI)	CCGGAATTCCATCCTGCTTGATGCGTTATGGA	52
albF-R (XhoI)	CCGCTCGAGGTGTCTTCTGAGCCTCCGATCAA	
Verification of MMA7 Δ*sbo-albF::cat* mutant		
ywiB-F	CACATGGAGTGTTATCGGTG	52
albFDown-R	CTGTAATCCGGTCCATGTGT	52
albA-R2	TGCCACAGTTTATGGACGAGAGG	52
albD-F	GCAGACAGAGCAGCAGCTCTGGA	52
cat255-R	CGTTTGTTGAACTAATGGGTG	52
cat958-D [#]	GGGTAACTAGCCTGCAGGCAATAGTTACCC	52
Bacteriocins screening primers		
Subtilin		
spaC-F	ACTATGAATCAATGGAAGG	48
spaS-R	TTGCAGTTACAAGTTAGTG	
Sublancin		
sublancin-F	GTGTGCTGCGTTGCTACAA	55
sublancin-R	TTGACGAGATACAAGCTAGTCC	
Subtilosin		
Sbo-F	GGTTGTGCAACATGCTCGAT	52
AlbA-R	CTCAGGAAGCTGGTGAACTC	
Subtilomycin		
subA2569-F	TGCGGATGACAGATTCGTATTGC	60
subA2571-R	ACAGCTGTACCGTGCCCATATAGA	

***** Introduced restriction sites are underlined; Cat, chloramphenicol resistance cassette; [#] [5].

Table S3. Sequence homology of the proteins encoded by the subtilomycin biosynthetic gene cluster *.

**Protein**	**No. aa**	**Closest homologue**	**% Identity/ (Aligned region aa)**	***E* Value** [#]
SubA	56	*Bacillus thuringiensis* serovar *thuringiensis* str. T01001.	56% (28/50)	3E−07
SubP	324	*Bacillus amyloliquefaciens* DSM 7 subtilisin-type proteinase	34% (113/333)	6E−44
SubB	1045	*Paenibacillus polymyxa* E681 lantibiotic biosynthesis protein SpaB	31% (336/1075)	6E−144
SubC	440	*Bacillus thuringiensis* IBL 200 Lantibiotic biosynthesis protein	44% (185/424)	8E−109
SubI	80	*Lactococcus* phage BK5-T hypothetical protein	31% (16/51)	1.3
SubT	585	*Staphylococcus epidermidis* VCU121 ABC transporter, ATP-binding protein	45% (258/573)	0.0

* Results are from a BLASTp search of the GenBank protein database on 17/04/2013; [#] Expectation value.

Figure S1. Mass spectrometry analysis of RP-HPLC purified peptide showing a number of oxidised forms typical of the thioethers found in lantibiotics.

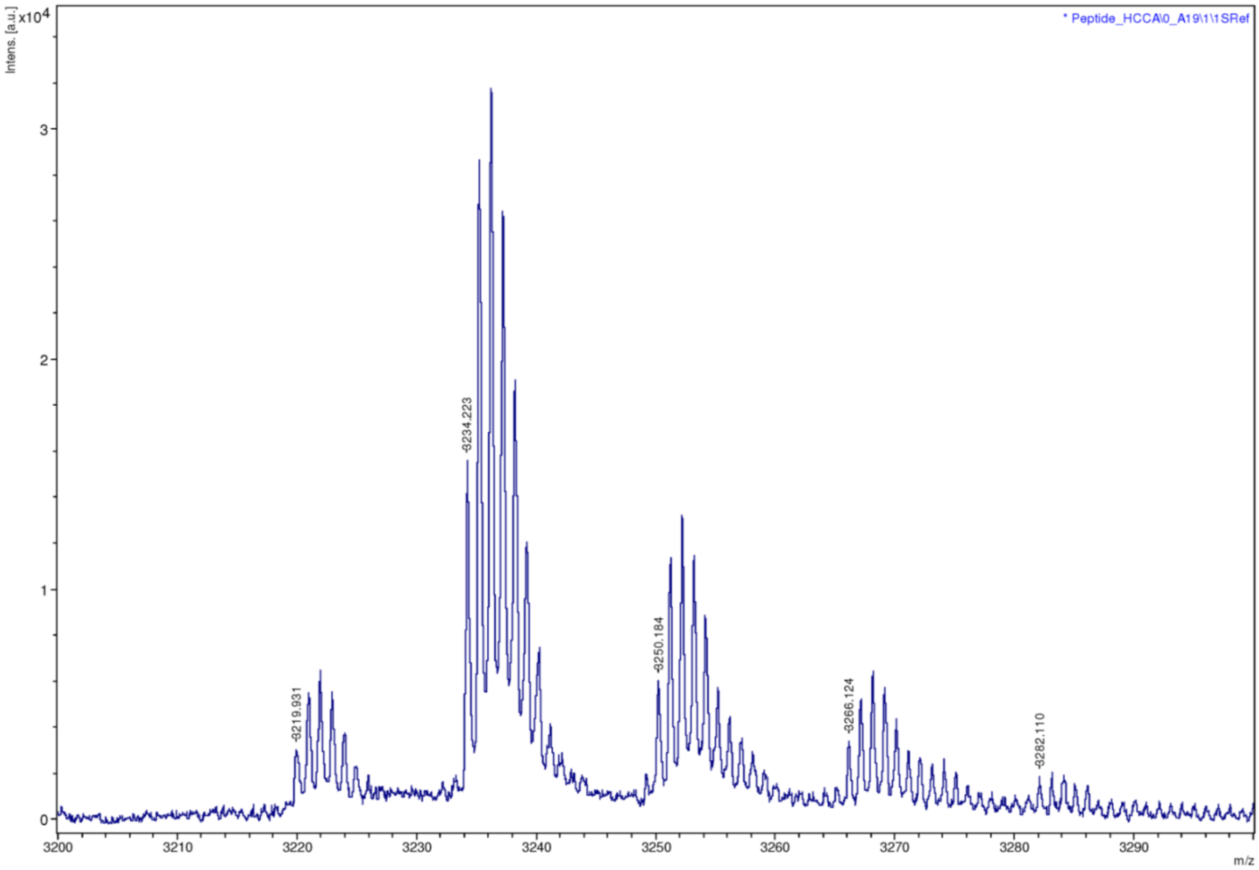

References

1. Phelan, R.W.; O'Halloran, J.A.; Kennedy, J.; Morrissey, J.P.; Dobson, A.D.; O'Gara, F.; Barbosa, T.M. Diversity and bioactive potential of endospore-forming bacteria cultured from the marine sponge *Haliclona simulans*. *J. Appl. Microbiol.* **2012**, *112*, 65–78.
2. Mac Aogain, M.; Mooij, M.J.; Adams, C.; Clair, J.; O'Gara, F. Emergence of extended-spectrum beta-lactamase and fluoroquinolone resistance genes among Irish multidrug-resistant isolates. *Diagn. Microbiol. Infect. Dis.* **2010**, *67*, 106–109.
3. Holden, M.T.; Seth-Smith, H.M.; Crossman, L.C.; Sebaihia, M.; Bentley, S.D.; Cerdeno-Tarraga, A.M.; Thomson, N.R.; Bason, N.; Quail, M.A.; Sharp, S. The genome of *Burkholderia cenocepacia* J2315, an epidemic pathogen of cystic fibrosis patients. *J. Bacteriol.* **2009**, *191*, 261–277.
4. Crossman, L.C.; Gould, V.C.; Dow, J.M.; Vernikos, G.S.; Okazaki, A.; Sebaihia, M.; Saunders, D.; Arrowsmith, C.; Carver, T.; Peters, N. The complete genome, comparative and functional analysis of *Stenotrophomonas maltophilia* reveals an organism heavily shielded by drug resistance determinants. *Genome Biol.* **2008**, *9*, R74.
5. Zilhao, R.; Serrano, M.; Isticato, R.; Ricca, E.; Moran, C.P.; Henriques, A.O. Interactions among CotB, CotG, and CotH during assembly of the *Bacillus subtilis* spore coat. *J. Bacteriol.* **2004**, *186*, 1110–1119.

© 2013 by the authors; licensee MDPI, Basel, Switzerland. This article is an open access article distributed under the terms and conditions of the Creative Commons Attribution license (http://creativecommons.org/licenses/by/3.0/).
